# When a pandemic and an epidemic collide: COVID-19, gut microbiota, and the double burden of malnutrition

**DOI:** 10.1186/s12916-021-01910-z

**Published:** 2021-01-28

**Authors:** Paula Littlejohn, B. Brett Finlay

**Affiliations:** 1 Department of Microbiology and Immunology, University of British Columbia, Vancouver, V6T 1Z3 Canada; 2 Michael Smith Laboratories, University of British Columbia, Vancouver, V6T 1Z4 Canada; 3 Department of Biochemistry and Molecular Biology, University of British Columbia, Vancouver, V6T 1Z3 Canada

**Keywords:** COVID-19, Gut microbiota, Double burden of malnutrition, Food insecurity

## Abstract

**Background:**

It is estimated that the COVID-19 pandemic will drastically increase all forms of malnutrition. Of particular concern, yet understated, is the potential to increase the double burden of malnutrition (DBM) epidemic. This coexistence of undernutrition together with overweight and obesity, or diet-related non-communicable disease (NCD), within low- to middle-income countries (LMICs) is increasing rapidly. Although multiple factors contribute to the DBM, food insecurity (FI) and gut microbiota dysbiosis play a crucial role. Both under- and overnutrition have been shown to be a consequence of food insecurity. The gut microbiota has also been recently implicated in playing a role in under- and overnutrition, with altered community structure and function common to both. The pandemic has already caused significant shifts in food availability which has immediate effects on the gut microbiome. In this opinion paper, we discuss how COVID-19 may indirectly exacerbate the DBM through food insecurity and the gut microbiome.

**Main text:**

The World Food Programme (WFP) estimates that 265 million people in LMICs will experience acute hunger in 2020 due to the pandemic, nearly doubling the original projection of 135 million. Global border closures to food trade, loss of food production, and stark decline in household income will exacerbate starvation while simultaneously necessitating that families resort to calorie-dense, nutrient-poor foods, thereby increasing obesity.

While food insecurity, which is the persistent lack of consistent access to adequate and nutrient-rich foods, will primarily drive nutrition behavior, the gut microbiome is perhaps a key biological mechanism. Numerous human and animal studies describe low diversity and an increase in inflammatory species as characteristic features of the undernourished and overnourished gut microbiota. Indeed, fecal transplant studies show that microbiota transfer from undernourished and overnourished humans to germ-free mice lacking a microbiome transfers the physical and metabolic phenotype, suggesting a causal role for the microbiota in under- and overnutrition. The observed microbiome dysbiosis within severe acute respiratory syndrome coronavirus 2 (SARS-CoV-2) coupled with the DBM presents a viscous cycle.

**Conclusion:**

Low- to mid-income countries will likely see an increase in the DBM epidemic. Providing access to nutritious foods and protecting individuals’ gut microbiome to “flatten the curve” of the DBM trajectory should be prioritized.

## Background

### Summary of the DBM epidemic

Malnutrition, in all its forms, is one of the leading underlying causes of morbidity and mortality worldwide [1]. According to the World Health Organization (WHO), approximately 462 million adults are underweight, 1.9 billion are overweight or obese, and 2 billion are micronutrient deficient. Among children under the age of 5, 144 million were stunted, 47 million wasted, 340 million micronutrient deficient, and 38.3 million considered overweight or obese in 2019 [2]. While an estimated 45% of deaths among children under the age of 5 each year are attributed to undernutrition, childhood overweight and obesity are also rising. This double burden of malnutrition is increasing at a 30% faster rate in children in many developing countries compared to high-income countries [3].

### Summary of the COVID-19 pandemic

The COVID-19 pandemic caused by the novel SARS-CoV-2 is an ongoing public health challenge. While vaccines are just becoming available, infection and death rates continue to climb [4]. As of December 2020, more than sixty-seven million people globally have been infected, and over 1.5 million deaths reported [4]. While North America currently leads with the highest number of cases (over fifteen million), there has been a sharp increase in South Asian, Latin American, and Caribbean countries [5]. At the time of this article, more than 36 million cases have been reported in the Americas, 27 million in Europe, 12 million in South Asia, 5 million in Eastern Mediterranean, 1.9 million in Africa, and over 1 million in the Western Pacific (WHO COVID-19 Dashboard). Among LMICs, India, Brazil, and Mexico have the highest number of cases [6]. These countries present with some of the highest prevalence of DBM, which are likely to increase during the pandemic. Smaller countries like Peru and Columbia are also experiencing significant increases in COVID-19 cases and deaths.

## How COVID-19 may impact food insecurity to exacerbate the DBM

COVID-19 is expected to exacerbate all forms of malnutrition worldwide, a detrimental setback to the Sustainable Development Goals (SDG2) to end all forms of malnutrition by 2030.

This is of grave concern, and indeed, a recent publication in *The Lancet* estimates that an additional 6 to 7 million children under the age of 5 will become wasted in 2020. They also project that an additional 10,000 children will die per month due to COVID-19-related factors without timely intervention. In September 2020, the WFP et al. reported that 370 million children have missed out on school meals due to COVID-19-related school closures [7]. This impacts children’s nutritional quality and quantity and predisposes them to become underweight or obese later in life by programming various metabolic pathways and tissues. According to a report from Save the Children, approximately 600 million children and families did not receive any government financial support due to COVID-19 [8]. There is concern that nutrition deterioration combined with governments’ social and physical restrictions to stop the virus’s spread is likely to impact obesity rates. Indeed, concerns around the potential increase in obesity rates have been expressed by the USA and UK [9, 10]. Low- to mid-income countries will likely not be protected from the same fate.

Numerous studies report that millions of families in LMICs have been pushed into poverty due to the pandemic, which has an immediate impact on malnutrition. Before the outbreak, 585 million children lived in monetary impoverished households; that number now sits at 702 million [8]. Surveys from several countries have already shown significant increases in food shortage among households with children directly linked to the pandemic [11–13]. Since the outbreak, 150 million additional children have been added to multidimensional poverty [8]. In addition to infection, COVID-19 also undermines nutrition and educational programs, food systems, healthcare, and humanitarian efforts in LMICs [14, 15]. Reports from the United Nations Children Funds (UNICEF) during the first half of the pandemic projects a sharp decline (75–100%) in maternal and child nutritional services during lockdown periods and a 30% decline overall when not in lockdown [14, 15]. These setbacks are likely to be felt for decades, and the road to recovery extended [6]. While multiple environmental and biological factors contribute to malnutrition, we focus on food insecurity and the gut microbiota as two mechanisms that will be impacted during COVID-19.

Forced border closures, the inability to import foods, and decreased production of fresh fruits and vegetables due to farm closures and worker shortages will exacerbate food insecurity and necessitate that families resort to calorie-dense, nutrient-poor foods, which have immediate negative impacts on weight and the gut microbiota. Food insecurity (i.e., the uncertainty over, the inability to access, and the lack of availability of adequate food) is equally felt by under- and overnourished individuals and countries of various income levels [11–13]. Whether moderate or severe, FI has been rapidly increasing over the last 6 years and affects one fourth of the global population. Recent data show that 50% of the people in Africa, about 30% in Latin America and the Caribbean, and over one fifth in Asia are food insecure [16].

Pre-COVID-19, 750 million people globally were exposed to severe food insecurity in 2019, and 2 billion did not have sufficient access to nutritious food, according to the *State of Food Security and Nutrition in the World* report [16]. Of these, 1.03 billion are in Asia, representing the highest burden, 205 million in Latin America and the Caribbean, 675 million in Africa, 88 million in North America and Europe, and 5.9 million in Oceania [16]. Moreover, 690 million were deemed to be chronically undernourished, roughly 8.9% of the world’s population. The report also suggests that several million more people will become undernourished in 2020 due to the pandemic, and child overweight and obesity will also likely increase.

The Global Report on Food Crisis (GRFC) also reported that in 2019 135 million people across 55 countries were in a food crisis. Although data is unavailable for all 55 countries, in September 2020, the GRFC reported that 101–105 million people across 27 of these countries spanning March–September 2020 were in a food crisis or worse [16]. Most importantly, areas with lower initial rates of food insecurity before COVID-19 will see the largest increases, and areas that were already food insecure will continue to have relatively higher rates. Disturbingly, 1 in 4 children may become food insecure in 2020 due to COVID-19. Both the USA and Canada report that food insecurity was higher in households where there were children under the age of 18 [13, 17]. This has implications for maternal child health as they are disproportionately affected by food insecurity.

There is consensus from the global nutrition community that the pandemic will plunge LMICs to extreme food crisis levels. This elevated level of food insecurity increases malnutrition, particularly among women and children, causing a relapse in gains made to date, making it highly unlikely to achieve Zero Hunger by 2030, a goal that was already off-target [14, 15]. The International Food Policy Research Institute (IFPRI) conservatively projects that children in over 100 LMICs will become wasted in 2020, and 128,605 will die due to the pandemic compared to recent pre-COVID-19 projections [14].

As previously mentioned, COVID-19’s disruption in food systems will cause some families to resort to inexpensive high-fat, low-nutrient foods. Recent analysis shows that dietary quality decreases with increasing levels of food insecurity [16]. Food-insecure people also consume less meat, dairy products, fruits, and vegetables than their food-secure counterparts [16]. These nutrients are critically essential for bone growth, immune protection, proper hormonal signaling, and gut microbiome stabilization. People living in LMICs have less diverse diets and primarily rely on diets rich in carbohydrates (e.g., rice and maize) and less on fruits and vegetables [16], a diet not conducive to optimal growth and development. UNICEF reports that less than 40% of infants and children meet the minimum recommended dietary diversity. Those in the poorest households are less likely to consume diets that meet at least five of the eight food groups. This type of low-diversity, high-carb diet is linked to gut dysbiosis and alterations in gut physical structure leading to NCDs possibly through insulin and leptin signaling pathways [18]. Moreover, diets low in fruits and vegetables lack the necessary micronutrients that act as co-factors in various metabolic pathways and fiber needed for healthy gut microbiota and host growth and development. Conservative estimates suggest that healthy diets will be five times more expensive in LMICs, which will be replaced by cheaper poor-quality foods, further reinforcing the negative impact on both the gut microbiota and DBM directly [16].

Although the association between FI and some forms of malnutrition shows mixed results, evidence demonstrates a direct relationship between food insecurity and undernutrition in children [19]. Similarly, a positive association between low birth weight and FI has been shown. An examination of eight LMICs (Bangladesh, Brazil, India, Nepal, Pakistan, Peru, South Africa, and the United Republic of Tanzania) in the Malnutrition and Consequences for Child Health (MAL-ED) study showed a positive association between food insecurity and stunting among children [20]. Mixed associations have been demonstrated between FI and wasting in children [19].

**Fig. 1:**
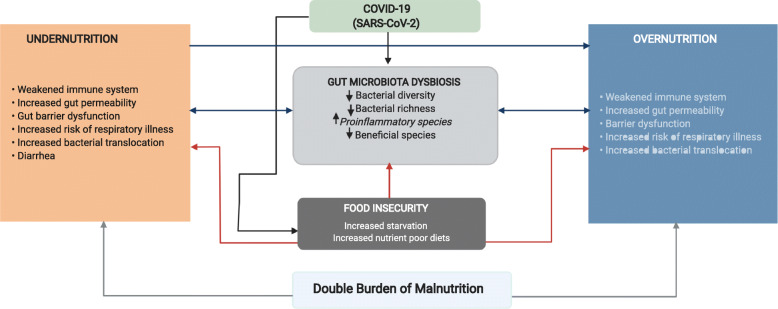
A proposed biological and environmental view of COVID-19 and DBM interaction. From the top: SARS-CoV-2 modulates the gut microbiota composition leading to reduced diversity, richness, and beneficial species and enrichment of proinflammatory species. DBM show similar gut microbiota signature and physiology, thus predisposing to SARS-CoV-2 severity. COVID-19 also impacts food insecurity driving both starvation and consumption of ultra-processed foods, resulting in leading to stunting, wasting, and obesity. This negative looped interaction impacts the gut microbiome and weight driving the double burden of malnutrition. Created with BioRender.com

Food insecurity does not only lead to undernutrition [21]. Indeed, even in ordinary times, food insecurity is directly associated with higher obesity prevalence, particularly among minority and lower-income groups [22]. A recent study found an association between severe food insecurity and the DBM in mothers and children (i.e., household level) in Brazil [23]. More than 42% of the mothers were overweight/obese, and 7.2% of children were stunted. Similar studies in India show an association between food insecurity, undernutrition, and dietary diversity [24]. The authors examined 2630 households in Maharashtra, India. They found that moderate and severe FI households were more likely to have stunted, severely underweight, and wasted children with lower dietary diversity scores. Dietary diversity was more strongly associated with stunting and underweight. An association between food insecurity and overweight in children is virtually absent. Associations between food insecurity and obesity among women, however, show positive results. The link between high-fat and sugary diets and obesity is well established. This type of diet has also been shown to induce gut microbiota dysbiosis and inflammation and alter host metabolism. Gut microbiota in obese has also been shown to harvest more energy. Figure 1 diagrams how COVID-19 interacts with food insecurity and the gut microbiome in the DBM.

## Human and animal studies of the role of the gut microbiota in malnutrition

While food insecurity will constitute the main environmental factor driving the DBM, the gut microbiome is at the core of the hosts’ metabolic response to diet that contributes to disease outcome. The causal role of the gut microbiome’s contribution to undernutrition and overweight/obesity is increasingly being appreciated, and multiple lines of evidence have examined these relationships [25–28]. Both conditions commonly present with gut microbiota dysbiosis, altered nutrient metabolism, and enrichment of proinflammatory species [29, 30].

To understand the gut microbiota’s causal role in undernutrition, Smith et al. examined Malawian twin pairs’ microbiota, 9 well-nourished and 13 discordant for kwashiorkor (severe acute malnutrition) at 3 years. They found that the microbiota in the discordant twin pairs failed to mature on a healthy trajectory compared to the well-nourished twin pairs, despite dietary intervention with ready-to-use therapeutic food (RUTF). The authors then transferred the fecal samples into gnotobiotic (i.e., germ-free) mice and found that the malnourished phenotype could be transferred. Mice receiving fecal microbiota transplant (FMT) from malnourished twins experienced significant growth faltering, both a decrease in anti-inflammatory and an increase in inflammatory bacterial species, than mice who received FMTs from well-nourished twins [31].

In a similar study, Blanton et al. transferred gut microbiota from 6- and 18-month-old healthy, severely stunted, and underweight Malawian infants into young germ-free mice fed a standard Malawian diet. Mice who received the undernourished microbiota experienced growth and metabolic impairment similar to their donors. Undernourished mice were then co-housed with healthy microbiota recipient mice, which transferred beneficial species to the undernourished mice that rescued growth abnormality.

Moreover, a recent meta-analysis showed that antibiotic use in children suffering from severe acute malnutrition in LMICs has growth-promoting effects [32]. Large intervention trials across multiple LMICs, however, seem to have conflicting results [29]. Nevertheless, these studies support a causal role of the gut microbiota in undernutrition.

Similar studies have been done to examine the overnourished microbiota in mice, and they describe a dysbiotic microbiota, loss of beneficial taxa, and enrichment of proinflammatory microbes to be a common “signature” [30, 33]. Although studies examining the link between the gut microbiota and overnutrition in humans are mainly correlative/associative, murine models repeatedly show a causative role. Germ-free mice do not progress to obesity when fed a high-fat diet (HFD); however, when colonized with bacteria from non-obese conventional mice and fed an HFD, they incur increased body and fat mass [34]. By transplanting obese adult fecal microbiota into germ-free mice, Ridaura et al. were able to determine a causal role of the gut microbiota in obesity. Further, co-housing the obese mice with lean mice prevented increased weight gain in the obese cage mates [35]. Other gnotobiotic models have reported similar results when microbiota from obese mice is transferred to lean or germ-free mice [26, 35, 36]. While the gastrointestinal tract of mice and gut microbiota composition is physiologically and structurally similar to that of humans in some regard, caution is needed to avoid overinterpretation of results [37]. Future improvement in gut microbiota research methodology and data interpretation tools will mitigate some of these challenges. Nevertheless, these data are convincing and currently being used to guide microbiota-targeted therapies.

What do we know about the gut microbiome and the DBM specifically? A recent study aimed to characterize the gut microbiota in DBM children. Méndez-Salazar et al. examined the gut microbiota of undernourished and overweight school-aged children in Mexico [38]. Using 16sRNA sequencing of fecal microbiota, the authors found that lower species richness and diversity was common to the undernourished (*n* = 12) and obese (*n* = 12) group compared to the controls (*n* = 12). A high abundance of *Bacteroides* was positively correlated with dietary fat in the obese group and carbohydrate in the undernourished group. This adds support to the similarities of both conditions though the gut microbiota may function differently. *Proteobacteria* were also overrepresented in the obese group, while *Firmicutes* and *Lachnospiraceae* dominated the undernourished gut. While the study sample size was modest, it nonetheless highlights the need for further studies to understand better the contribution of the gut microbiota in the DBM.

Research on the impact of food insecurity on the gut microbiome is sparse. However, a recent pilot study examined the gut microbiome in 3-month-old infants from food-insecure homes and found that they had an altered gut microbiome than those from food-secure families [39]. Further research is warranted to validate these results. A recent publication by Christian et al., November 2020, reviewed the impact of FI on malnutrition and gut microbiome and reported that the gut microbiota is changed in a food-deprived state. The authors pointed to gut microbiota dysbiosis and immaturity as a consequence of FI that underlies malnutrition during the pandemic [40].

## COVID-19 alters gut microbiota: implications for the DBM

To date, a growing number of studies have investigated the role of the gut microbiota and SARS-CoV-2. A recent shotgun metagenomics sequencing from a small cohort of 15 COVID-19 patients revealed significant gut microbiota dysbiosis in patients versus controls. The authors observed an increase in the abundance of pathogenic bacteria and decreased beneficial microbes, which persisted even after resolving respiratory symptoms and negative throat swabs [41]. The authors found an association between several species, namely *Coprobacillus*, *Clostridium ramosum*, and *Clostridium hathewayi*, and more COVID-19 disease severity [41]. Further, species involved in short-chain fatty acid production and anti-inflammatory capacity were negatively associated with COVID-19 severity. The authors found several *Bacteroides* species (*B. dorei*, *B. thetaiotamicron*, *B. massiliensis*, and *B. ovatus*) to be inversely correlated with viral load. Interestingly, these species were also noted to downregulate the ACE2 receptor in the gut [41]. Similarly, Gu et al. investigated the gut microbiota of 30 patients with COVID-19. Results showed a significantly lower Shannon and Chao diversity index indicative of lower alpha diversity (species diversity within a community) and richness (total number of species) in COVID-19 patients compared to controls. Overall community structure differed significantly between COVID-19 patients and controls (beta-diversity metrics). Additionally, the authors found that *Proteobacteria* (an inflammatory species) dominated the gut of COVID-19 patients [42]. Compared to the healthy controls, COVID-19 patients had a dramatically reduced abundance of the families *Ruminococcaceae* and *Lachnospiraceae*, which is also observed in malnourished individuals. A significant association between microbiota composition and severity of COVID-19 was not found [42]. Various researchers have proposed a role for the gut microbiota in COVID-19 pathogenesis and as a potential therapeutic target [41, 43–46]. Indeed, probiotics and prebiotics have been proposed as possible prevention and treatment of COVID-19 [47]. Large human cohort studies, as well as gnotobiotic animal models, are needed to tease apart these relationships.

In addition to acting as an immune barrier, the gut microbiota also confers significant nutritional benefits to the host, such as fermentation of indigestible fibers to make short-chain fatty acids (SCFAs), biosynthesis of B vitamins and vitamin K, and nutrient absorption [48]. As discussed above, undernourished human cohorts and mouse models have shown that metabolic function is disturbed due to altered microbiota and that this abnormal metabolic phenotype could be transferred [25]. Likewise, microbiota from obese mice shows an increased energy harvest capacity, which is transferrable to germ-free mice [26, 34].

SARS-CoV-2 appears to modulate gut microbiota structure; thus, shifting it to a dysbiotic state could have adverse metabolic consequences. Our opinion that this coupled with food insecurity may further alter the gut microbiota’s functionality exacerbating malnutrition phenotypes.

## Future landscape: impact of COVID-19 during early life affects future DBM

Considerable evidence exists that nutrient deprivation during food shortage has long-term health effects, as seen in the Dutch and Chinese famine studies [49, 50]. These studies found that fetuses exposed to famine in early gestation experienced a greater prevalence of overweight and obesity and non-communicable disease later in life [49, 50]. In line with this, the Developmental Origins of Health and Disease (DOHaD) hypothesis, an adaptation to the Barker “Fetal Origin of Health and Disease” hypothesis, postulates that undernutrition during critical developmental windows (i.e., first 1000 days) contribute to adverse short- and long-term health outcomes [51–53]. The first 1000 days of life, the time between conception and the child’s first 2 years, is deemed a critical window in life where noxious environmental exposures can have adverse intergenerational effects [54, 55]. Interestingly, the first 1000 days is also a crucial window for the gut microbiome assembly, whereby perturbation to this process also carries health consequences later in life [56]. It has been proposed that the trajectory of gut microbiota of the offspring might be established before birth and that maternal nutrition and microbiota composition influences colonization [57–60]. Multiple studies have described the influence of maternal obesity on the infant’s microbiome at birth and in childhood [30, 59, 61]. Environmental and intrinsic inputs such as diet, infection, and stress, during this critical period, influence the microbial, metabolic, and immunological programming of the fetus and alter immediate and long-term health, including brain development and linear growth [54, 58, 59, 62].

Food insecurity also harms maternal and child nutrition. This population is reportedly more vulnerable to food crisis [16]. Pregnant women fearing the risk of COVID-19 may also access antenatal care much less, which puts them at high risk for pregnancy complications [63]. A recent study showed a significant decrease in antenatal care usage among rural pregnant Mayan women in Guatemala. The reduction was linked to fear of infection and limited staff availability [63].

Other unrealized impacts are the increase in low birth weight (LBW) and preterm infants due to restricted nutrition, both of which show an increased risk of NCDs later in life. Pregnant women, nursing mothers, and infants require adequate nutrition, which will be significantly lost during this time. Increased use of Caesarean sections may increase, which has been shown to affect the gut microbiota assembly, which, coupled with poor nutrition, will have a devastating health impact. A recent study in pregnant women in Wuhan at the start of the pandemic showed that COVID-19-positive women had an increased risk of both preterm birth and Caesarean sections compared to non-positive women [64]. However, no difference in birth weight was observed, and no infants tested positive for the virus.

As previously mentioned, food insecurity during critical windows negatively impacts linear growth and gut microbiota. Early nutrition, including breastfeeding, profoundly affects the gut microbiome’s assembly, and a healthy gut microbiome in children confers lifelong health benefits. Breastfeeding has been actively promoted in LMICs to mediate the effects of malnutrition and the DBM [65]. Interruption to these programs due to COVID-19, coupled with an inadequate diet, may cause lactating moms to cease nursing and introduce alternative feeding practices earlier, which will dramatically affect the establishment of beneficial microbes within the infant’s microbiome and influence linear growth and weight gain [29, 55, 56, 66]. Further, resource-poor countries shifting attention to COVID-19 will drastically reduce humanitarian programs and routine medical care, including prenatal care and vaccines, thereby potentially missing opportunities to provide and treat non-life-threatening illnesses, increasing infection burden, and contributing further to changes in the gut microbiota and susceptibility to SARS-CoV-2 [67].

## Conclusion

Considerable evidence exists that nutrient deprivation in times of food shortage has long-lasting health consequences. It is feasible that with the current pandemic within LMICs, we will see an increase in undernutrition and obesity (i.e., the double burden of malnutrition) both in the immediate future and in years to come. Multiple risk factors, such as food insecurity, gut microbiome, and related social determinants of health, contribute to all forms of malnutrition. Although LMICs have achieved much progress in reducing malnutrition, these countries remain fragile. Many of their gains will be lost due to the unexpected disturbances of COVID-19. Here we have described how food insecurity and gut microbiota links to the DBM and how this may be exacerbated within the context of the COVID-19 pandemic. Actions to mitigate some of these effects are sorely needed.

We recommend that LMICs aiming to flatten the curve of the DBM should prioritize efforts on nutrition. Increasing breastfeeding practices and access to low-cost nutritious foods, already demonstrated interventions, is paramount. Further, as a healthy gut microbiota modulates disease beyond the gut, targeting the microbiota may have unrealized benefits in the struggle against malnutrition. The use of probiotics and prebiotics to modulate the gut microbiota is well established. Leveraging these insights may prove effective in mitigating damage to the gut microbiome, which impacts malnutrition. An unexplored intervention would be to expedite community-level use of microbiota-directed complementary foods (MDCF), a diet designed by Gehrig et al. to repair and strengthen the microbiota. MDCF was shown to be an effective therapy to restore metabolic function and structure of the microbiome in Bangladesh children [68]. Combining these strategies may prove useful in the current pandemic to alter the trajectory of the DBM.

## Data Availability

Not applicable

## Abbreviations

ACE-2: Angiotensin-converting enzyme receptor-2
COVID-19: Coronavirus disease of 2019
DBM: Double burden of malnutrition
DOHaD: Developmental Origins of Health and Disease
FAO: Food Agricultural Organization
FMT: Fecal microbiota transplant
GRFC: Global Report on Food Crisis
HFD: High-fat diet
IFPRI: International Food Policy Research Institute
LBW: Low birth weight
LMICs: Low- to middle-income countries
MDCF: Microbiota-directed complementary foods
NCD: Non-communicable disease
RUTF: Ready-to-use therapeutic food
SARS-CoV-2: Severe acute respiratory syndrome coronavirus 2
UNICEF: United Nations Children Funds
WHO: World Health Organization
WFP: World Food Programme
